# Fine-Tuning a Transformer Model for METTL3 Lead Optimization

**DOI:** 10.1021/acsbiomedchemau.5c00198

**Published:** 2026-01-20

**Authors:** Christian M. Matter, Amedeo Caflisch

**Affiliations:** Department of Biochemistry, University of Zurich, CH-8057 Zurich, Switzerland

**Keywords:** machine learning, medicinal chemistry optimization, epitranscriptomics, METTL3, metabolic stability, UZH2

## Abstract

Transformers are machine learning models originally developed to translate between natural languages. Recently, a transformer model was trained on knowledge of medicinal chemistry, i.e., matched molecular pairs of nearly a million bioactive compounds from the ChEMBL database. Here, we customize (i.e., fine-tune) the pretrained model to enhance the affinity and/or metabolic stability of a series of inhibitors of methyltransferase-like protein 3 (METTL3). We first fine-tune the transformer model using a data set of about 500 METTL3 inhibitors with known binding affinities and validate it by retrospective analysis. Then, we fine-tune the original transformer model to simultaneously optimize binding affinity and metabolic stability in a prospective application. Two of the five METTL3 inhibitors predicted by the multiobjective optimized model show low-nanomolar potency and higher stability than the lead compound of the chemical series used for fine-tuning.

## Graphical abstract

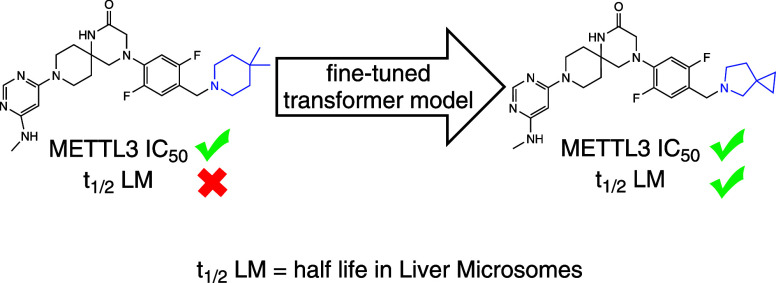

## 1 Introduction

The use of machine learning (ML) in drug discovery is evolving rapidly.^1,2^ Generative models are often used in the hit discovery and/or hit-to-lead phases of a drug discovery campaign. These models leverage huge training data sets to learn favorable interactions and frequent patterns in drug-like molecules. A much larger portion of the chemical space can be explored with such models than with classical chemical libraries.^1^

Chemical language models, which are inspired by the successful advance of natural language processing, are one of the ML tools used for the de novo generation of molecules. These models work with one-dimensional string representations of molecules, mostly SMILES^3^ or SELFIES.^4^ While SMILES are the most common one-dimensional representation in cheminformatics, they suffer from the fact that a large fraction of SMILES strings do not represent valid molecules. The SELFIES representation, on the other hand, was designed such that each SELFIES string represents a valid molecule.^4^ SELFIES are often preferred in chemical language models as the syntax is less demanding. With the same compute budget this leaves more time during the model training to learn the interesting molecular properties instead of the underlying language syntax. Both SMILES and SELFIES share the property that one molecule can be encoded by multiple different strings. Recurrent neural networks (RNN) with memory cells, such as long short-term memory (LSTM),^5^ and transformer architectures are in widespread use among chemical language models.^1,6^

In the hit-to-lead phase of drug discovery several properties have to be optimized simultaneously. These include compound potency, ADMET properties (absorption, distribution, metabolism, excretion and toxicity) and selectivity among other attributes.^7^ During lead optimization different derivatives of the same compound are synthesized and tested. As the main interactions between the target and the lead compound are already established, changes to the core of the lead compound are infrequent in this phase. The available data and number of tested compounds in lead optimization are usually too scarce to fully train chemical language models. To use these models for lead optimization, transfer learning^8^ approaches must be leveraged. Fine-tuning is a transfer learning approach in which a model that was pretrained on a similar task without prohibitive data limitations is trained further with the limited data set.^1^ From the pretraining the model should keep the basic rules such as chemical validity and frequent chemical patterns in the generated molecules. With the fine-tuning data set the chemical space to be sampled is reduced such that patterns in the fine-tuning data set should also be recovered in the generated molecules.

The methyltransferase-like protein 3 (METTL3) forms a heterodimeric complex with METTL14. In this complex METTL3 is the catalytic subunit which uses S-adenosylmethionine (SAM) as the methyl donor to methylate the amine group of RNA adenine bases at position 6.^9,10^ The resulting N^6^-methyladenosine (m^6^A) is the most prevalent mRNA modification in eukaryotes.^11^ This modification has been shown to regulate the processing,^12,13^ stability^14^ and translation^15,16^ of mRNA. There is accumulating evidence that abnormal levels of m^6^A modifications can lead to certain types of blood cancer, e.g., acute myeloid leukemia (AML)^17^ and solid tumors. The SAM-competitive METTL3 inhibitor STC-15 is in phase 1 clinical trials.^18^ Furthermore, SAM-competitive inhibitors with single-digit nanomolar potency in biochemical assays have been published by our group at the University of Zurich (the lead compound UZH2)^19^ and two pharmaceutical companies.^20,21^

Here, we fine-tune and apply a transformer model to improve the metabolic stability of UZH2. The original transformer model was developed for hit expansion and was trained on a large set of matched molecular pairs of bioactive molecules.^22^ We first fine-tune the model with a data set of binding affinity values of METTL3 inhibitors and validate it by a retrospective analysis. Then we fine-tune the transformer model to optimize both affinity and metabolic stability of the UZH2 series. The present study is focused on a single, highly congeneric METTL3 inhibitor series. In the Conclusions section we discuss potential extensions and clarify which observations might be valid in broader applications.

## 2 Methods

For further optimization of the UZH2 series of METTL3 inhibitors, we decided to fine-tune a previously published transformer model originally trained on pairs of similar bioactive molecules from the ChEMBL data set.^22^ The base transformer model was built as a tool for hit expansion and designed to learn frequent medicinal chemistry transformations of molecules, the most frequent being methylation, fluorination, and chlorination. The base model was generated by OpenNMT^23^ (opennmt-py, version 2.3.0) using nearly one million molecules in the ChEMBL database^24^ (version 28). The molecules in this data set were canonicalized and all stereochemical information was removed with RDKit^25^ (version 2023.03.3). The molecules were then fragmented and the fragments were paired based on common substructures using mmpdb.^26^ For training one molecule of the pair is taken as the input and the other molecule as the desired output. For the base model each pair was used twice with each of the molecules once being the input and once being the target molecule. The full training of the base model according to the procedure of Tysinger et al.^22^ is described in the Supporting Information (Figures S1 and S2).

The data preparation for the fine-tuning follows mostly the same procedure as for the base model with some important adjustments described in the next subsection. Unless otherwise stated, all fine-tuning runs started from epoch 10 of the base model as the perplexity score^27^ did not improve after the 10th epoch. This finding is consistent with the results reported in the original paper of the base model.^22^ The fine-tuning lasted for an additional 30 epochs with a learning rate of 1 and a batch size of 128. For model inference, nucleus random sampling^28^ with a probability of 0.9 was used. This means that the next item in the SELFIES sequence (i.e., SELFIES token which describes the next atom or bond such as [C], [N], [Ring], or [Branch]) was sampled only from the smallest possible set of high-probability candidates whose cumulative probability exceeds 0.9. An overview of the fine-tuning procedure is shown in Figure 1.

**Figure 1:**
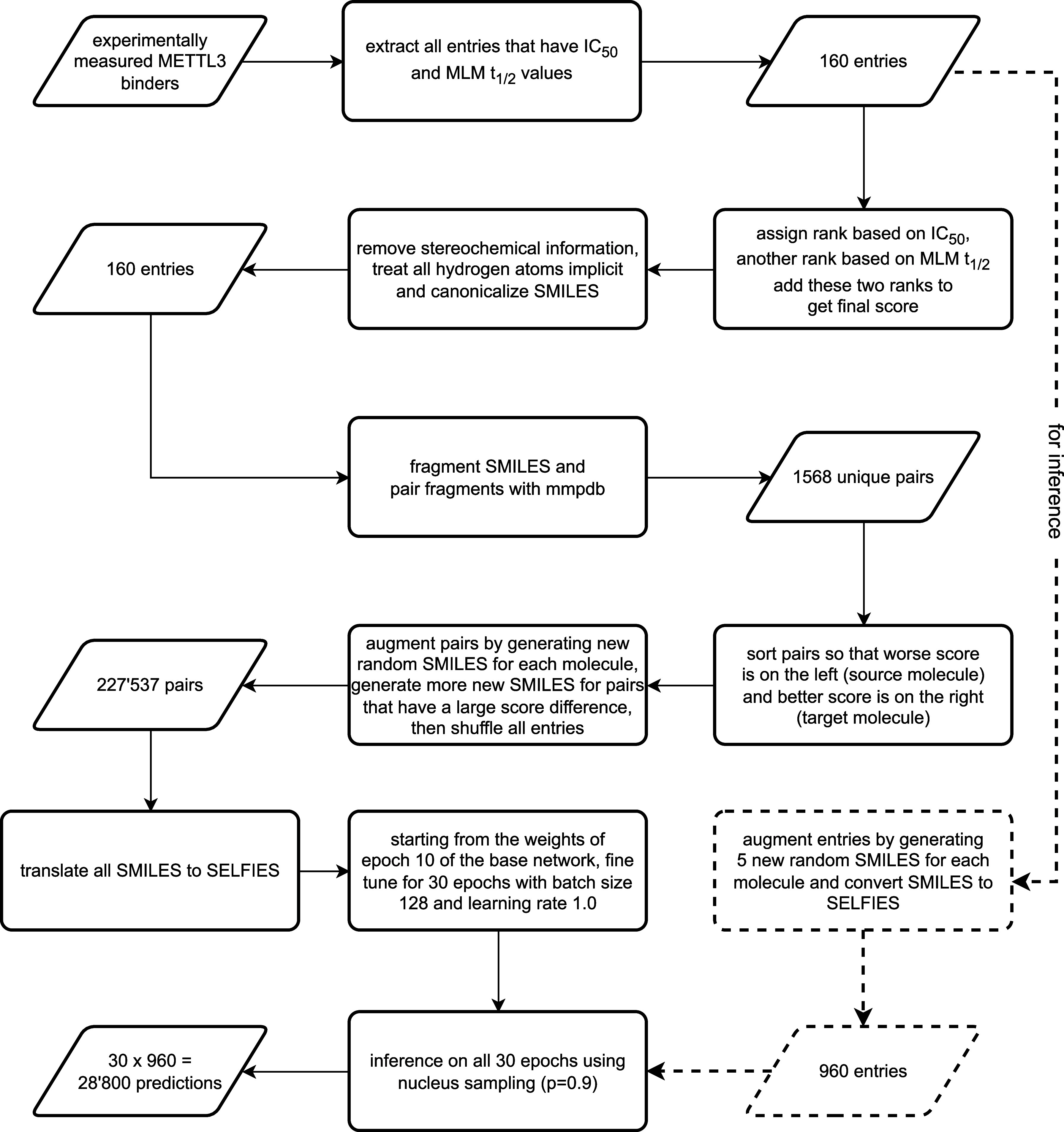
Schematic overview of the fine-tuning process. All numeric values refer to the multiobjective fine-tuning application mentioned in the results. MLM = mouse liver microsome.

### 2.1 Pair Ordering

As mentioned above, in the base model each pair of molecules is present twice in the training set. For fine-tuning we do not want the model to learn all transformations, but only those that improve one or more properties of the METTL3 inhibitors. By ordering each pair of molecules so that the worse molecule of the pair is considered as the input and the better molecule as the target, the model should only learn transformations that improve the chosen property.

The properties for sorting the molecules can be chosen freely. The only requirement is that they can be used to rank the molecules. Here, first the compound potency and later a score incorporating both the compound potency and metabolic stability were used for sorting. Ties in the sorting score were broken by using both pair orderings as in the base model training.

### 2.2 Augmentation

Compared to the nearly one million molecules in the data set for the training of the base model, only small data sets of 554 and 160 METTL3 inhibitors were available for the two independent fine-tunings, respectively. By augmenting the SMILES pairs, more data can be generated for fine-tuning. The augmentation takes advantage of the fact that multiple different SMILES strings can encode the same molecule. This property also holds when the SMILES are translated to SELFIES, with which the model operates. Random SMILES strings for the same molecule were generated with RDKit.^25^

The augmentation is done after the molecules are paired as the pairing based on substructure does not rely on SMILES strings and duplicate molecules would need to be removed. After augmentation the order between the different pairs was randomized such that during the fine-tuning the model encountered diverse pairs in each batch. The simplest augmentation method generates for each molecule of every pair the same number of random SMILES strings.

Augmentation can also be steered by favoring pairs with a large difference in score more than pairs with a small score difference. This reduces in the training set the fraction of pairs whose molecules have similar score. Transformations that lead to small score differences should be encountered less often by the model, as the improvement of the molecule is less significant. Pairs with a large score difference should be preferentially learned by the model and therefore encountered more often in the training set. The number of augmentations for each pair was calculated as

1 $$n_{i}=\lfloor \frac{d_{i}-d_{\min}}{d_{\max}-d_{\min}}\cdot n\rfloor$$

where *n*_*i*_ is the number of augmentations for pair *i*, ⌊·⌋ denotes the floor function, *d*_*i*_ is the score difference between the molecules of pair *i*, *d*_min_ and *d*_max_ are the smallest and largest score difference, respectively, and *n* scales the number of augmentations. Here *n* = 200 was used.

### 2.3 Experimental Section

#### 2.3.1 Mouse Liver Microsome Assay

The metabolic stability has been evaluated in a mouse liver microsome assay by BioDuro (Shanghai, China) following standard procedures. Briefly, the compounds (200 μM solution in DMSO) were incubated at 37 °C with mouse liver microsomes in a phosphate buffer solution (pH = 7.4). At several time points (0, 5, 15, 30, and 60 min), the internal standard in acetonitrile was added to the corresponding well to stop the reaction. The samples were vortexed vigorously for about 1 min and then centrifuged for 15 min (4000 rpm), and the supernatants were analyzed by LC–MS/MS.

#### 2.3.2 Enzymatic Assay

Inhibition of the enzymatic activity of METTL3 was measured by an assay based on time-resolved Förster resonance energy transfer (TR-FRET). The assay uses the m^6^A reader protein in the detection step as originally reported.^29^ The same assay was employed for the development of the UZH2 series^19^ and another series of METTL3 inhibitors.^30^

## 3 Results

We first present the results of fine-tuning using data on inhibitory activity, which is a retrospective study. We then discuss the simultaneous optimization of potency and metabolic stability (measured in liver microsomes) in a prospective application.

### 3.1 Fine-Tuning for Potency

Before using the fine-tuned model in a prospective study, we decided to test its ability to predict potent METTL3 inhibitors. The complete data set, originating from our medicinal chemistry campaign, consisted of 554 molecules with one or more moieties similar or identical to UZH2. For each molecule, the data set contained the SMILES string and the experimentally determined value of IC_50_ (inhibitor concentration that results in 50% reduction of signal with respect to the buffer) as measured by the TR-FRET assay. The IC_50_ of the 66 inactive molecules was set to 1 mM. This value is higher than any IC_50_ measured by the enzymatic TR-FRET assay. The top 20 molecules according to IC_50_ were removed as a holdout data set. Their IC_50_ values range from 1 to 5 nM. From the bottom 40% of the data set, 30 molecules (of which 10 were inactive) were extracted for inference by the fine-tuned model. We wanted to evaluate if the model can predict unknown potent molecules from also unknown inactive molecules or molecules with low potency. This left 504 molecules for the actual fine-tuning (Figure S3).

After fragmenting, pairing, and ordering the molecules based on IC_50_ the pairs were augmented 30 times by generating different random SMILES representations for the molecules. These were converted to SELFIES and used for fine-tuning. After fine-tuning the previously excluded 30 molecules for inference were augmented 20 times in the same manner as the training molecules and used to predict new molecules from each of the 30 fine-tuning epochs. A total of 6841 unique molecules were predicted in this retrospective study.

Of the 20 molecules in the hold out set, five were predicted at least once, and more precisely 6, 4, 2, 2, and 1 times, respectively. Two representative predictions of a hold out molecule can be seen in Figure 2. Most of the transformations from input to the predicted molecule occurred in the substituents, and only occasionally was the scaffold changed. This behavior is likely due to the fact that most of the active molecules in the training set share the same spiro scaffold as UZH2.

**Figure 2:**
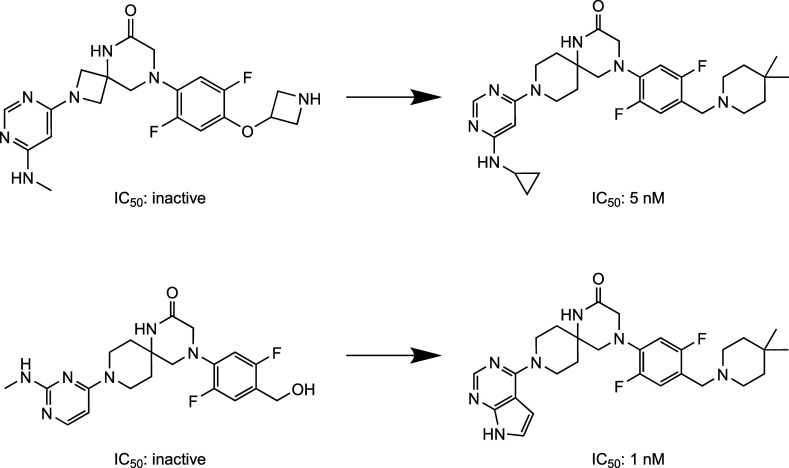
Example transformations of the fine-tuned model. (Left) Input molecule given to the fine-tuned transformer model. (Right) Molecule predicted by the fine-tuned transformer model. The predicted molecules are part of the hold-out set that the transformer model has not seen during training.

To further assess the quality of the predictions, the compound most similar to each predicted molecule was identified in the experimentally measured data set. For identifying the closest molecule, the Tanimoto similarity using Morgan fingerprints with a radius of two was used. Each predicted molecule was assigned the IC_50_ value of the closest known molecule if the similarity was 0.8 or greater. The resulting distribution is shifted toward higher potency (lower IC_50_ values) compared to the distribution of experimentally measured IC_50_ values (Figure 3). The threshold of 0.8 is a compromise between a significant number of predictions and a high similarity to known compounds. A similar shift toward higher potency was observed with similarity thresholds of 0.75, 0.85, and 0.90 (Figure S4). As desired, there are few predictions that are similar to the least potent or known inactive compounds. Thus, the fine-tuned transformer model predicts molecules similar to the top inhibitors more frequently than molecules similar to weakly active compounds. As the transformer model is fine-tuned for this specific chemical series, the enrichment toward known active chemical neighborhoods will probably not hold when predicting molecules far from the training data. But as mentioned above, it can predict highly potent molecules of the hold out set it has never before encountered as long as the chemical structure is similar.

**Figure 3:**
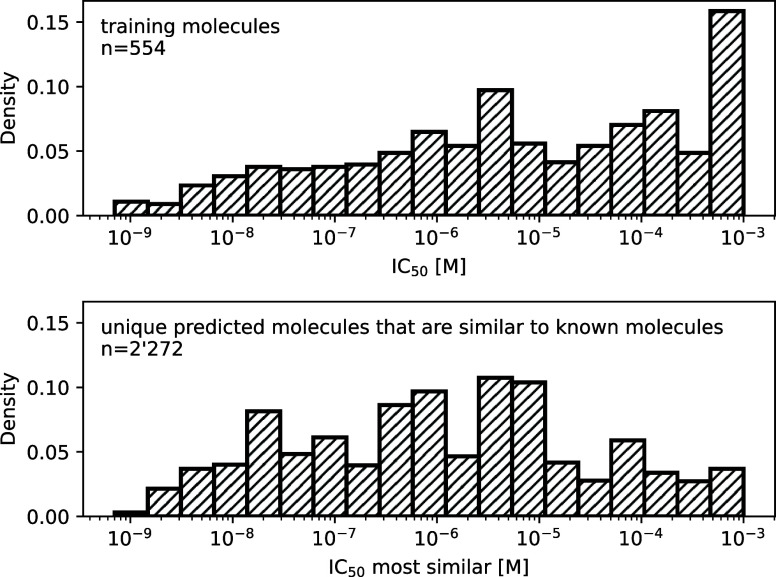
Comparison of predicted molecules and training set. (Top) Distribution of the IC_50_ values for all experimentally measured molecules. (Bottom) For all unique predicted molecules that have a Tanimoto similarity of at least 0.8 to a known molecule, the IC_50_ of the known molecule was used for the histogram. The bar at IC_50_ = 10^–3^ M represents inactive molecules.

### 3.2 Multiobjective Fine-Tuning

The goal of this prospective study was to predict new inhibitors of METTL3 with improved metabolic stability while maintaining low-nanomolar potency. A data set of 160 molecules with experimentally measured inhibitory activity values in the enzymatic assay (IC_50_) and half-life values (*t*_1/2_) of metabolic stability in mouse liver microsomes (MLM) was used for the fine-tuning of the base model. The lead compound UZH2 ^19^ was not in the training set as its metabolic stability was measured in rat liver microsomes (RLM) and human liver microsomes (HLM) but not in MLM. However, the HLM and RLM data were available only for a few compounds, and thus MLM stability was employed for the fine-tuning. A score incorporating both potency and MLM stability was calculated for each molecule by ranking the 160 molecules separately by potency and metabolic stability. For each molecule, the sum of the two ranks was used as the final score. The unweighted sum is a simple model which does not require any additional parameter. Alternative weighting schemes might be used at different stages of lead optimization (see Conclusions). As in the single-objective study, the IC_50_ of the experimentally measured molecules found to be inactive was set to 1 mM, which is higher than any measured IC_50_. After fragmentation and pairing, 1568 pairs were generated from the 160 molecules mentioned above. Each pair of molecules was first sorted according to the sum of the two ranks and then augmented by generating different SMILES strings for the molecules. Pairs with a high score difference between the constituent molecules were augmented a higher number of times than pairs with a low score difference according to eq 1. Here *n* = 200 was chosen which resulted in 227 536 pairs after augmentation.

After fine-tuning, all 160 SMILES of the data set were augmented five times to be used for inference. This time, the augmentation was done without any bias from the score. With these augmented SMILES strings new molecules were predicted for each of the 30 fine-tuning epochs. There were 2824 unique molecules among the 28 800 predictions. The 2824 unique predictions were first reduced to 2379 by PAINS^31^ filtering with an established filter list.^32^ The remaining 2379 molecules were further reduced to about 300 by selecting for novel groups and small scaffold changes compared to UZH2. Especially the predictions from the first epochs of the fine-tuning contained a lot of molecules which were very dissimilar to UZH2 and therefore discarded. Subsequently, visual inspection was used to eliminate about 90% of the predictions. This selection was further reduced to five compounds according to the estimated synthetic accessibility. The molecules **P1**–**P5** were then synthesized and their potency and metabolic stability were determined experimentally (Figure 4). The purity of the compounds was larger than 95%. For compounds **P3** and **P4** the racemic mixture was synthesized and tested.

**Figure 4:**
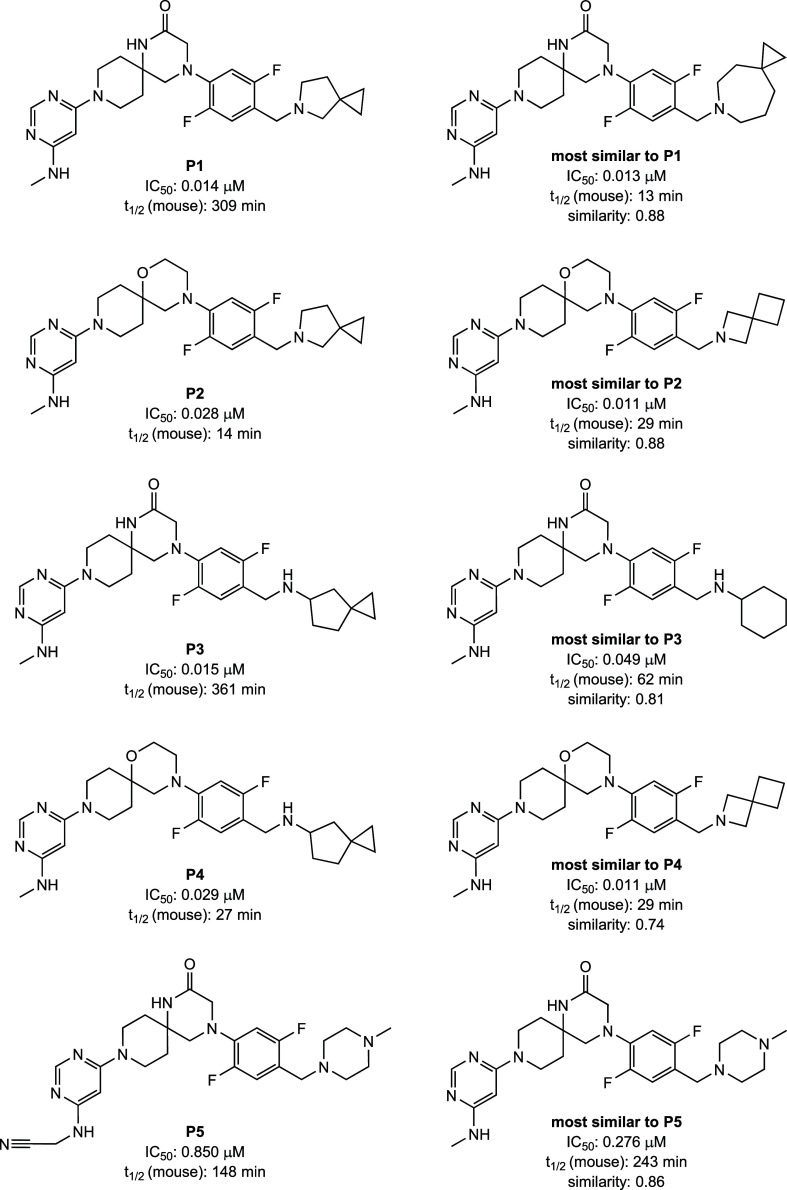
(Left) The five new predicted molecules that were synthesized. (Right) The most similar molecule in the training set. The values for compounds **P3** and **P4** refer to their racemic mixtures.

In a first analysis, we checked if **P1**–**P5** could have been generated by just applying the transformation rules extracted from the ChEMBL data set and the fine-tuning data set. There are a total of 18 613 100 different SMIRK transformations stemming from the ChEMBL data set and 1537 different SMIRK transformations from the fine-tuning data set. 361 SMIRKs from the ChEMBL data set involve 5-azaspiro[2.4]­heptane (**P1** and **P2**) with the N-substitution. A few of those SMIRKs could generate **P1** or **P2**, respectively, starting from a molecule in the fine-tuning data set. One such transformation that is captured in the ChEMBL SMIRKs is from the molecule **most similar to P2** to **P2** (see Figure 4). In the ChEMBL data there are only seven SMIRKs involving spiro[2.4]­heptane with the linking as in **P3** and **P4**. None of these would lead to **P3** or **P4** when applied to the fine-tuning molecules. There are many more transformations in the ChEMBL data set leading to 2-aminoacetonitrile (**P5**), with some of them generating **P5** when applied to the fine-tuning data set. Looking at the SMIRK transformations extracted from the fine-tuning data set, the closest transformation to **P1**–**P4** involves 6-azaspiro[2.6]­nonane (see **most similar to P1** in Figure 4). For **P5** there are SMIRKs involving 2-aminoacetonitrile but none of them would lead to **P5** when starting from any of the fine-tuning training set molecules. It is important to note that for all the predictions **P1**–**P5** the input molecule to the transformer model was more than one SMIRK away from the prediction. So none of the transformer predictions were captured one-to-one in the ChEMBL or fine-tuning SMIRKs.

When applying all SMIRK transformations to all training molecules in a purely combinatorial approach, **P3** and **P4** would not be generated. **P1**, **P2** and **P5** could be generated but they would be drowned in the vast quantity of predicted molecules, most of which would not improve the desired properties. In contrast, the fine-tuned transformer model prioritizes the SMIRKs. Moreover, it can employ entirely new transformations and apply multiple transformations in one step for improving the properties of the predictions.

It is good practice to compare the predicted molecules to the closest ones in the training set.^33^ For each predicted molecule, the most similar molecule in the training set was identified using Tanimoto similarity acting on Morgan fingerprints with a radius of two, which is equivalent to ECFP4 fingerprints (Figure 4).

Compared to their most similar compound in the training set, prediction **P1** achieved the same potency while significantly increasing the half-life value (*t*_1/2_) of metabolic stability in MLM from 13 to 309 min. Compound **P3** improved both the potency and MLM stability compared to its closest training set molecule. In contrast, the predicted compounds **P2**, **P4**, and **P5** have a slightly lower potency and metabolic stability than their most similar compounds in the training set.

It is interesting to compare the predicted molecules with the lead compound UZH2,^19^ which was not part of the training set as mentioned above. UZH2 was the most frequently predicted molecule (2971 times, i.e., about 10%) while **P1** to **P5** were predicted 3, 1, 2, 1, and 2 times, respectively. For **P1–P4** the slightly reduced potency (factor 3 to 6) results in ligand efficiency (LE) similar to UZH2 (Table 1). In contrast, **P5** shows a substantial loss of potency. In general, the best predictions are compounds **P1** and **P3** which have a slightly poorer potency (factor of 3) than UZH2, and a higher and similar HLM stability, respectively. Their MLM stability is about 5 and 6 h, respectively, which is substantially higher than for the closest compounds in the training set (Figure 4). Moreover, the RLM stability of compound **P1** is nearly 1 h which is significantly higher than for UZH2 (24 min).

**Table 1:** Five Synthesized Molecules Originating from Fine-Tuning Compared to UZH2

compound	IC_50_ ^a^ (nM)	MW^b^ (g·mol^–1^)	LE^c^	HLM^d^ (min)	RLM^e^ (min)	MLM^f^ (min)
UZH2^g^	5	514	0.31	4.5	24	–
**P1**	14	498	0.30	9.2	57	309
**P2**	28	485	0.30	5.6	–	14
**P3**	15	512	0.29	4.8	–	361
**P4**	29	499	0.29	7.5	–	27
**P5**	850	526	0.22	–	–	148

a FRET-based assay.

b Molecular weight.

c Ligand efficiency (kcal·mol^–1^·heavy atom count^–1^).

d Human liver microsomes, *t*_1/2_.

e Rat liver microsomes, *t*_1/2_.

f Mouse liver microsomes, *t*_1/2_.

g Data from Dolbois et al.^19^ The values for compounds **P3** and **P4** refer to their racemic mixtures.

After the comparison of the predicted inhibitors **P1–P5** with the closest molecules in the training set (Figure 4) and with the lead compound UZH2 (Table 1), we now focus on a comparison with the entire training set of 160 molecules (Figure 5). The predicted compounds **P1** and **P3** populate the most favorable sector of the scatter plot of potency vs MLM stability. Moreover, they rank first and second (of 165 molecules), respectively, according to the score used for fine-tuning (sum of MLM and IC_50_ ranks), and thus represent an improvement with respect to the training set. In contrast, the predicted compound **P5** ranks 37, which is mainly due to its high nanomolar affinity.

**Figure 5:**
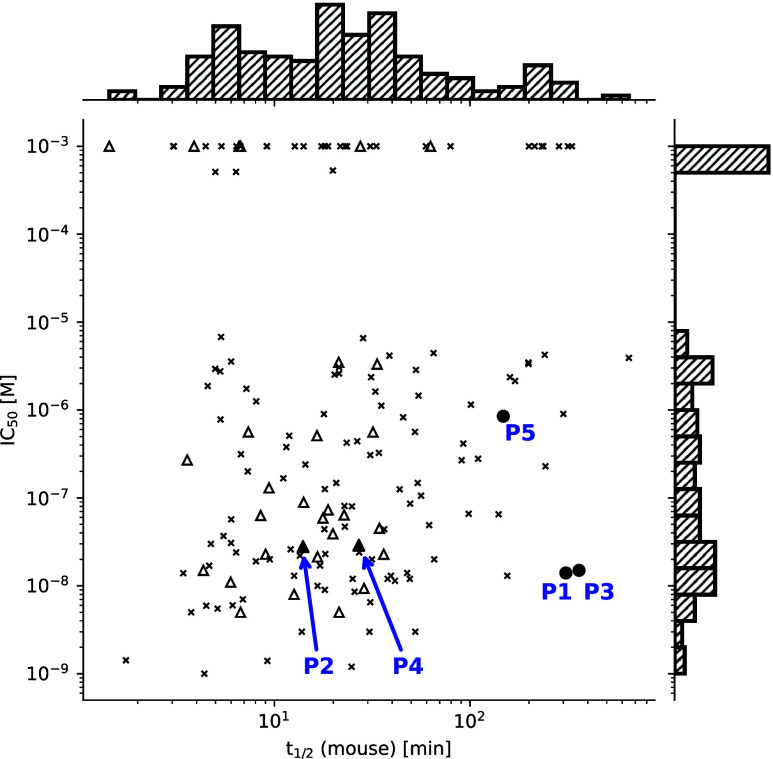
Scatter plot of potency and metabolic stability in mouse liver microsome (MLM) for the 160 molecules in the training set (crosses and empty triangles) and the five predicted molecules (filled circles and filled triangles). Triangles represent molecules with a spiro-morpholine scaffold. Data points at IC_50_ = 10^–3^ M represent inactive molecules.

For the two predictions with the spiro-morpholine scaffold (compounds **P2** and **P4**), it is more adequate to compare them with the training set compounds that feature the same scaffold (29 molecules, empty triangles in Figure 5). Both predictions with the spiro-morpholine scaffold have above-average potency and/or MLM stability. Compounds **P4** and **P2** rank 5 and 14, respectively, among the 29 + 2 compounds with spiro-morpholine scaffold in the training set. Furthermore, compound **P4** is one of the most stable spiro-morpholine compounds.

Overall, the model fine-tuned on potency and metabolic stability made useful predictions (compounds **P1** and **P3**) but suggested also uninteresting derivatives (**P5**) of the single-digit nanomolar lead UZH2. The mixed predictive ability is not surprising as it is usually difficult to improve the potency of low nanomolar compounds, and it is even more challenging to further improve both potency and metabolic stability.

## 4 Conclusions

We have fine-tuned a transformer model for further improvement of a series of METTL3 inhibitors. The original model published by Tysinger et al. in 2023 contains the most frequent modifications of medicinal chemistry (e.g., methylation, fluorination, and chlorination) as it was trained on matched molecular pairs of a library of nearly one million bioactive molecules in the ChEMBL database.^22^ The novel aspect of our work is the model calibration, i.e., the fine-tuning of the medicinal chemistry-trained transformer for further optimization of an advanced series of inhibitors. In a metaphoric picture, the fine-tuning presented here corresponds to a machine translator from a foreign language to Italian that is further optimized for a given lexicon, e.g., the verbiage of the romance *The Leopard* by Tomasi di Lampedusa. Such model calibration results in translations into Italian sentences with an abundance of terms related to nobility and its decline as in *The Leopard*.

There are two main observations from the two independent fine-tunings, respectively. First, the model fine-tuned by a set of about 500 METTL3 inhibitors is able to predict molecules similar to the top inhibitors more frequently than molecules similar to weakly active compounds. Furthermore, it is able to predict some of the single-digit nanomolar inhibitors of the hold-out set it has never before encountered. Second, in the prospective application, the model fine-tuned on both potency and metabolic stability data has predicted two new METTL3 inhibitors with low-nanomolar potency and metabolic stability of several hours in mouse liver microsomes.

One critical question remains outstanding. Is a fine-tuned transformer more efficient in improving an advanced series of inhibitors than a skilled medicinal chemist? It is not possible to answer this question here as we focused on a single series of compounds for a single enzyme target. Moreover, medicinal chemistry intuition was used for the final selection of five compounds from the predictions of the model. Thus, (fine-tuned) transformer models might be more useful as decision-support tools than autonomous optimizer. We consider our work a pilot study which should spur the attention of research groups in pharmaceutical companies that have the means for a statistically significant comparative assessment on multiple chemical series and/or protein targets.

Concerning future extensions, the use of Group SELFIES^34^ might improve the quality of the predicted molecules compared to the original SELFIES strings. Another possible extension is the use of publicly available liver microsome (or hepatocyte assays) and/or cell-permeability data (e.g., Caco-2 cells) to further train the base-model with the knowledge of metabolic stability and/or cellular uptake. Future applications might consider a score based on combinations of any properties given the flexibility of the multiobjective optimization. Furthermore, multiplicative factors could be used to weight different properties, e.g., in the final optimization stage of an advanced series one could assign higher weight to ADME properties than potency. Here we selected the simplest score with equal weight on metabolic stability and inhibitory activity. For the development of chemical probes it might be more adequate to use a score based on potency and cell-permeability rather than metabolic stability which is essential only for in vivo experiments.

The presented fine-tuning procedure and applications did not use any physics-based method. Synergistic combinations of machine learning tools and physics-based methods are expected to be superior for projects in which only a few ligands are available, e.g., at the start of a hit optimization campaign.^35^ Moreover, physics-based methods can be used to alleviate a main limitation of (fine-tuned) transformer models which is the lack of prioritization of the predictions. To improve a weakly active hit compound into a potent lead, one can consider a force field-based postprocessing of the molecules predicted by the (fine-tuned) transformer model. High-throughput docking into a crystal structure (or a protein structure generated by deep learning) with an efficient evaluation of the binding energy^36–39^ could be used for postprocessing.

## Supplementary Material







## Notes

Training of the base transformer model used for fine-tuning and expanded fine-tuning results (PDF)
IC_50_ values used for potency fine-tuning (CSV)
IC_50_ and *t*_1/2_ values used for multiobjective fine-tuning (CSV)
