# Supplementary material for: Fine-Tuning a Transformer Model for METTL3 Lead Optimization
Source: ACS Bio Med Chem Au. 2026 Jan 20;6(2):160–7. doi: 10.1021/acsbiomedchemau.5c00198 (PMC13087808; doi:10.1021/acsbiomedchemau.5c00198)
Supplement: Supplementary file 1 [file bg5c00198_si_001.pdf]

Supporting Information:

Fine-Tuning a Transformer Model for METTL3 Lead Optimization

Christian M. Matter and Amedeo Caflisch*

Department of Biochemistry, University of Zurich, CH-8057 Zurich, Switzerland

E-mail: caflisch@bioc.uzh.ch

Base Model

In this section the training of the base model according to Tysinger et al.[S1] is described. Because their weights are not in the public domain, they were generated ab initio. A schematic overview of the training can be seen in Figure S1.

From the ChEMBL[S2] data set (version 28) all entries whose 'molecule type' was 'small molecule' and the entry contained an IC_{50}, EC_{50} or K_i value were extracted. All salts were removed by checking if the SMILES string of the entry contains a dot. This left a total of 940 640 entries. The SMILES strings of these entries were transformed such that they are canonical, do not contain any stereochemical information, and all hydrogen atoms are treated implicit. These transformations were done with RDKit.[S3] After removing all SMILES that could not be converted to SELFIES,[S4] the SMILES were fragmented and the fragments were paired with mmpdb.[S5] This resulted in 29 596 204 pairs after duplicate pairs were removed.

The SMIRK transformation[S6] between the molecules of each pair was recorded and the frequency of each SMIRK was determined. To capture relevant SMIRKs, all pairs that had a SMIRK that occurred less than 50 times in the whole data set were excluded. Additionally, to prevent that a few most frequent SMIRKS (i.e., methylation, fluorination, and chlorination) dominate the data set, only 50 pairs were sampled randomly for each SMIRK. This resulted in 11 738 SMIRKs with 50 pairs each for a total of 586 900 pairs.

A train, validation, test split of the pairs was done using the following chronological splits: If the ChEMBL entry date of both molecules in the pair was before 2013, they were added to the training set. If the entry date of both molecules was between 2013 and 2015 including, they were added to the validation set. All remaining pairs were added to the test set. For each pair the corresponding pair with flipped order of molecules was also added to the train, validation, or test set respectively. This resulted in 596 850 pairs in the training set, 304 346 pairs in the validation set and 272 604 pairs in the test set. All SMILES were converted to SELFIES before being used for training, validation, or testing.

For training, the default model architecture from OpenNMT[S7] (opennmt-py, version

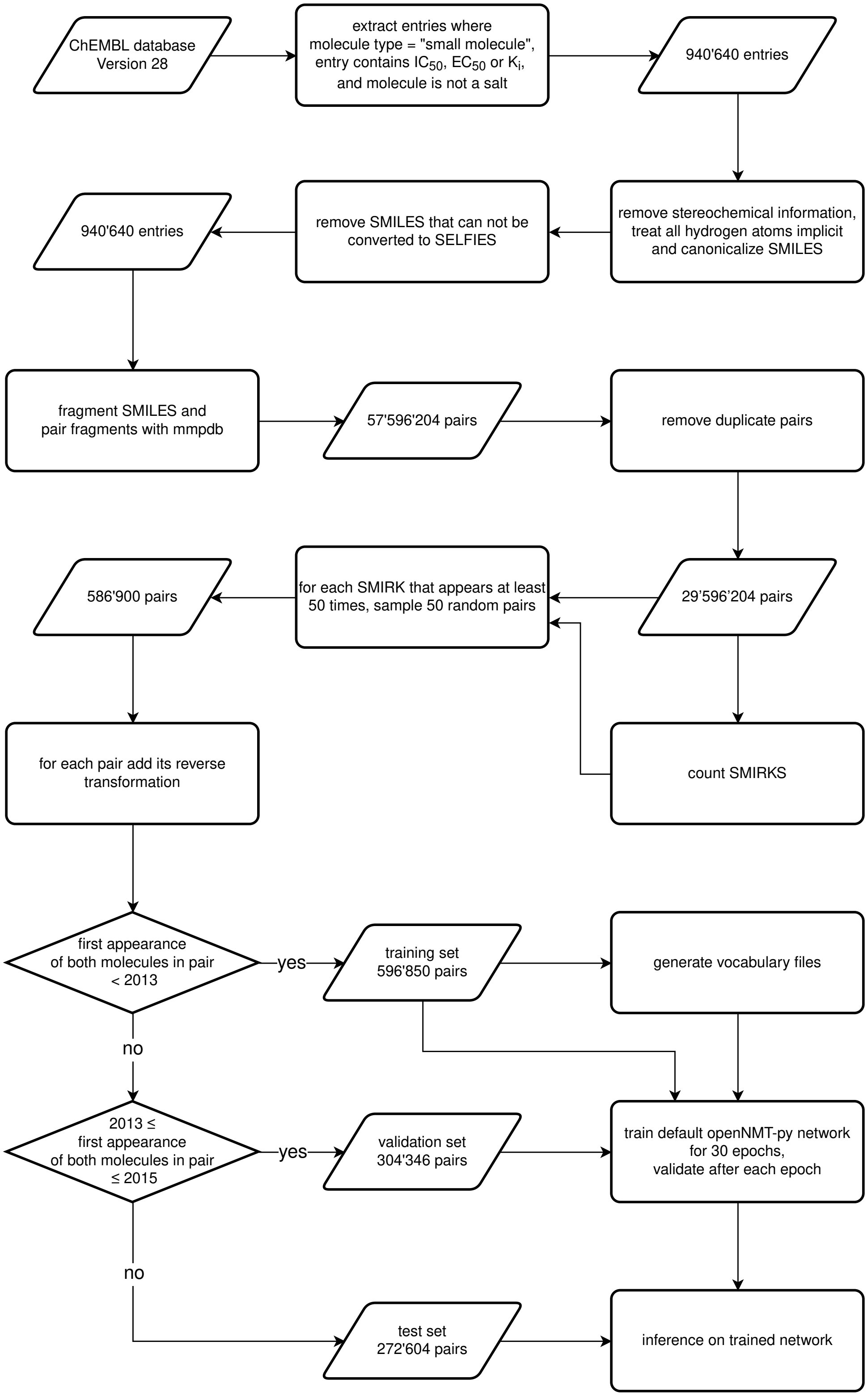

Figure S1: Schematic overview of the training procedure for the base sequence-to-sequence transformer model.

2.3.0) was used with its default parameters. Vocabulary files, which contain all the different SELFIES tokens that appear in the training set, were also generated with OpenNMT. The model was trained for 30 epochs with a batch size of 128 and a learning rate of 1.0. After each epoch the model weights were saved and the validation set was used for inference.

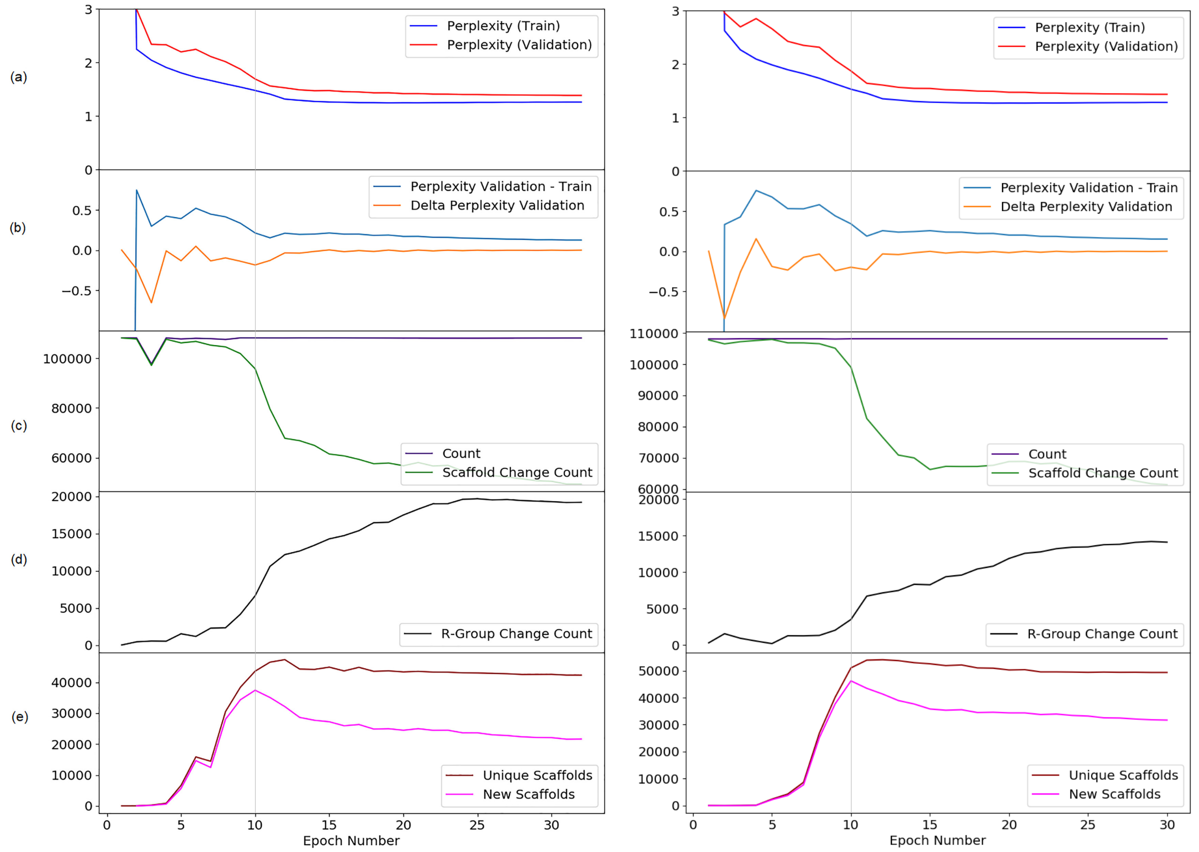

Figure S2: Comparison of the base transformer model[S1] (left column) and our re-training (right column). In (a) the training and validation perplexity of the model is shown. In (b) the difference between the perplexity of the validation and the training set is visualized, together with the difference in the perplexity of the validation set compared with itself from the previous epoch. (c) shows the total number of predicted molecules, and how many of these new predictions have a new scaffold compared to the molecule used as an input for this prediction. The number of molecules for which only an R-group changed compared to the corresponding input molecule is shown in (d). In (e) the number of unique scaffolds and the number of new scaffolds in the predictions are shown. The vertical gray lines mark epoch 10 which was used as the starting point for the fine-tuning.

In Figure S2 multiple metrics of the model during the training progress are shown. Comparing the original model from Tysinger et al. to our retraining, we observe that the overall

trends over the epochs are very similar. As expected the perplexity of both the training and the validation set decreases over the epochs. In the first 10 epochs the validation perplexity is much more erratic than in the later epochs. In both models the validation perplexity gets close to the training perplexity towards the higher epoch numbers. The scaffold change count is stable for the first epochs and then falls drastically between epoch 8 and 15 for both models. The difference between the scaffold change count for the earlier epochs compared with the later epochs is a bit higher for the model of Tysinger et al. After epoch 15 the scaffold change count is again more stable with a small downwards trend after epoch 22. At the same time as the scaffold change count drops, the R-group change count rises sharply. This is to be expected as each prediction is classified into either scaffold change, R-group change or no change. As the model learns, less drastic changes are made to the input molecules. The scaffold change count falls and the R-group change count rises. In our model the R-group change count does not quite reach the same height as in the model of Tysinger et al., mostly because the scaffold change count does not drop as low in our model as for the model of Tysinger et al. Lastly, the number of unique and new scaffolds is close to zero for the early epochs because the model repeats itself and predicts almost the same molecule no matter the input molecule. As the model learns to adapt to the input molecule the number of unique and new scaffolds rises until a certain point, when the scaffold change count starts to drop again. The modifications are now mostly on the R-groups and the scaffold is hardly changed any more. This explains the small decrease in unique scaffolds, as some of the input molecules have the same scaffold, and the bigger drop in new scaffolds as only R-group changes do not produce a new scaffold.

In the magnitude and to a lower extent in the shape of the above discussed metrics over the epochs there are some differences between the original model[S1] and our re-traning. Overall these differences are rather small and could easily stem from the randomness in the composition of the training set or the model weight initialization. The initial ChEMBL database extract used was the same for both models. The data set was sampled according

to SMIRKS as described above. This sampling led to different final training, validation, and test sets for the two models.

Epoch 10 of our re-trained model (vertical gray line in Figure S2, right) was used as a starting point for the fine-tuning.

Fine-tuning for Potency

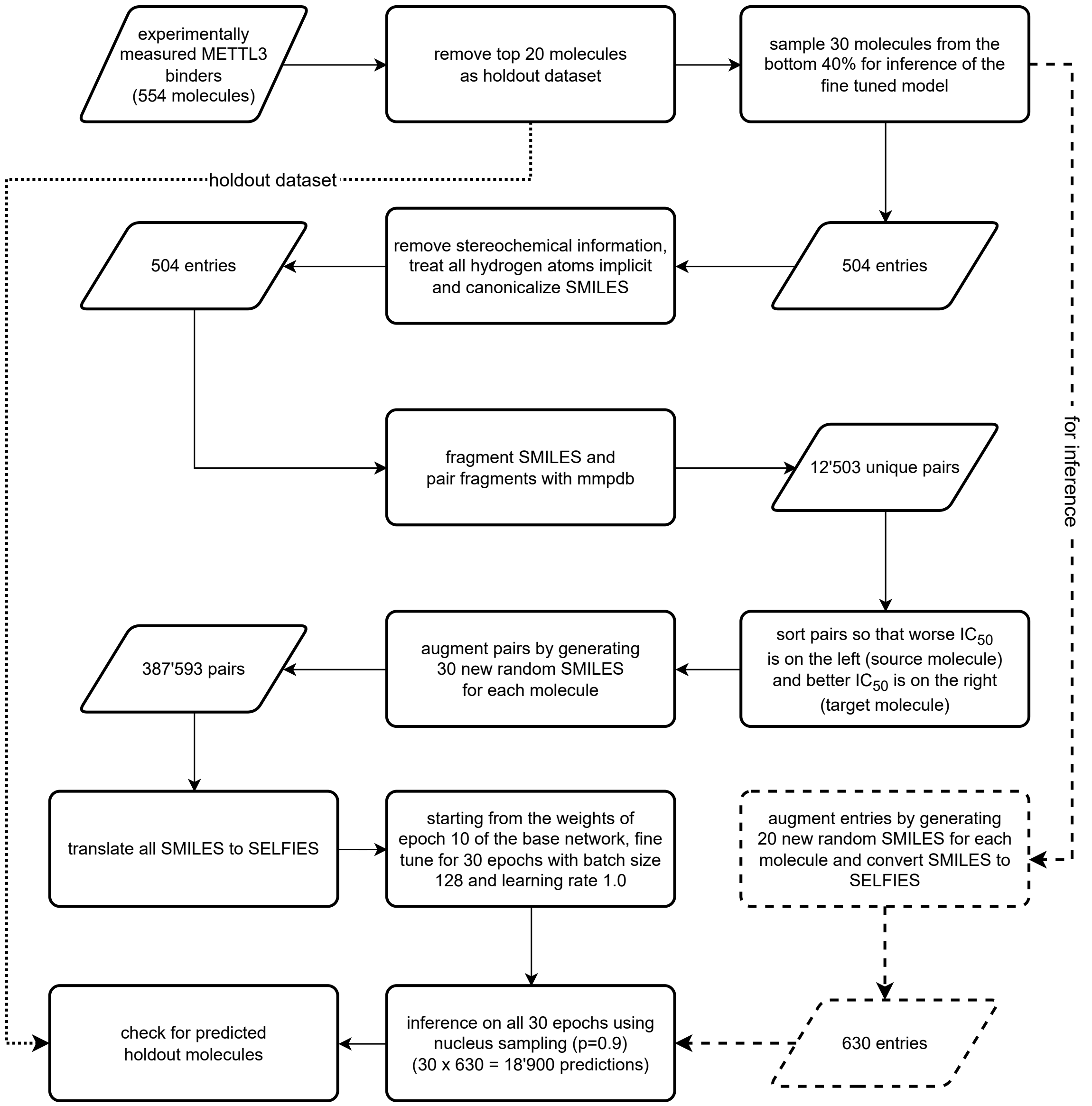

Figure S3: Schematic overview of the fine-tuning process for potency.

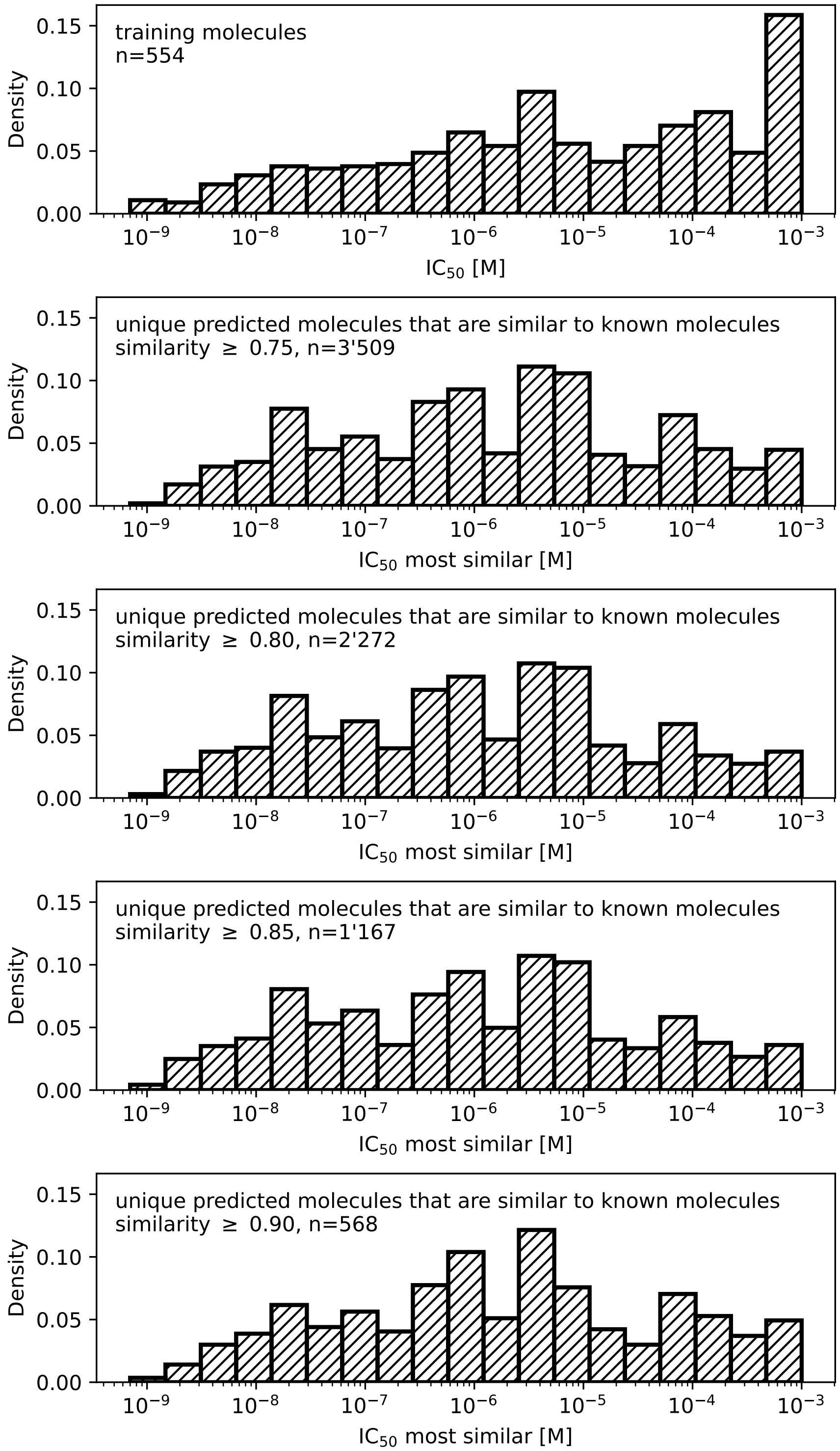

Figure S4: Robustness of the distributions of IC_{50} values with respect to the threshold used for Tanimoto similarity. (Top) Distribution of the IC_{50} values for all experimentally measured molecules. (Other panels) For all unique predicted molecules that have a Tanimoto similarity equal or bigger than the threshold to a known molecule, the IC_{50} of the known molecule was used for the histogram. The number of unique known molecules are 403, 385, 365 and 354 for the panels with similarity $\geq$ 0.75, 0.80, 0.85, and 0.9, respectively. The bar at $IC_{50} = 10^{-3}$M represents inactive molecules.

References

(S1) Tysinger, E. P.; Rai, B. K.; Sinitskiy, A. V. Can We Quickly Learn to "Translate" Bioactive Molecules with Transformer Models? *J. Chem. Inf. Model.* **2023**, *63*, 1734–1744.

(S2) Mendez, D.; Gaulton, A.; Bento, A. P.; Chambers, J.; De Veij, M.; Félix, E.; Magariños, M. P.; Mosquera, J. F.; Mutowo, P.; Nowotka, M.; Gordillo-Marañón, M.; Hunter, F.; Junco, L.; Mugumbate, G.; Rodriguez-Lopez, M.; Atkinson, F.; Bosc, N.; Radoux, C. J.; Segura-Cabrera, A.; Hersey, A.; Leach, A. R. ChEMBL: towards direct deposition of bioassay data. *Nucleic Acids Res.* **2018**, *47*, D930–D940.

(S3) RDKit: Open-source cheminformatics. https://www.rdkit.org, Version 2023.03.3, DOI: 10.5281/zenodo.8254217.

(S4) Krenn, M.; Häse, F.; Nigam, A.; Friederich, P.; Aspuru-Guzik, A. Self-referencing embedded strings (SELFIES): A 100% robust molecular string representation. *Mach. Learn.: Sci. Technol.* **2020**, *1*, 045024.

(S5) Dalke, A.; Hert, J.; Kramer, C. mmpdb: An Open-Source Matched Molecular Pair Platform for Large Multiproperty Data Sets. *J. Chem. Inf. Model.* **2018**, *58*, 902–910.

(S6) Daylight. SMIRKS: A Reaction Transform Language [Internet] [cited 2025 May 05]. http://www.daylight.com/dayhtml/doc/theory/theory.smirks.html.

(S7) Klein, G.; Kim, Y.; Deng, Y.; Senellart, J.; Rush, A. OpenNMT: Open-Source Toolkit for Neural Machine Translation. Proceedings of ACL 2017, System Demonstrations. Vancouver, Canada, 2017; pp 67–72.
